# The barriers and facilitators to implementing dementia education and training in health and social care services: a mixed-methods study

**DOI:** 10.1186/s12913-020-05382-4

**Published:** 2020-06-05

**Authors:** Claire A. Surr, Sahdia Parveen, Sarah J. Smith, Michelle Drury, Cara Sass, Sarah Burden, Jan Oyebode

**Affiliations:** 1 Centre for Dementia Research, Leeds Beckett University, School of Health and Community Studies, Leeds, LS1 3HE UK; 2 Centre for Applied Dementia Studies, University of Bradford, Bradford, UK

**Keywords:** Alzheimer’s disease, Behaviour change, Care homes, Education, Hospitals, Mental health services, Training, Workforce development

## Abstract

**Background:**

The health and social care workforce requires access to appropriate education and training to provide quality care for people with dementia. Success of a training programme depends on staff ability to put their learning into practice through behaviour change. This study aimed to investigate the barriers and facilitators to implementation of dementia education and training in health and social care services using the Theoretical Domains Framework (TDF) and COM-B model of behaviour change.

**Methods:**

A mixed-methods design. Participants were dementia training leads**,** training facilitators, managers and staff who had attended training who worked in UK care homes, acute hospitals, mental health services and primary care settings. Methods were an online audit of care and training providers, online survey of trained staff and individual/group interviews with organisational training leads, training facilitators, staff who had attended dementia training and managers. Data were analysed using descriptive statistics and thematic template analysis.

**Results:**

Barriers and facilitators were analysed according the COM-B domains. “Capability” factors were not perceived as a significant barrier to training implementation. Factors which supported staff capability included the use of interactive face-to-face training, and training that was relevant to their role. Factors that increased staff “motivation” included skilled facilitation of training, trainees’ desire to learn and the provision of incentives (e.g. attendance during paid working hours, badges/certifications). “Opportunity” factors were most prevalent with lack of resources (time, financial, staffing and environmental) being the biggest perceived barrier to training implementation. The presence or not of external support from families and internal factors such as the organisational culture and its supportiveness of good dementia care and training implementation were also influential.

**Conclusions:**

A wide range of factors may present as barriers to or facilitators of dementia training implementation and behaviour change for staff. These should be considered by health and social care providers in the context of dementia training design and delivery in order to maximise potential for implementation.

## Background

To deliver person-centred care for people with dementia, staff across health and social care services require knowledge, skills and empathic attitudes [1]. The provision of good quality dementia education and training can assist in achieving this [2]. While there is an expansive literature on development and efficacy of dementia education and training interventions [3], there remains limited evidence about the barriers and facilitators to implementing learning in practice.

Research indicates that education alone is not enough to lead to staff behaviour change and the transfer of learning into daily practice [4]. However, few studies have considered systematically the barriers and facilitators to implementing dementia training and staff behaviour change, and none have examined commonalities over a range of setting types, services and training programmes. The studies that have explored this area identify organisational and service factors such as peer support, mentorship and supervision [5, 6], alignment of the training with the organisation’s care culture and senior/leadership support [7] as important facilitators for effective training.

Implementation theory research examines the theoretical underpinnings of behaviour and behaviour change. The Theoretical Domains Framework (TDF) [8] is a widely used, integrative framework of theories of behaviour change that can be used to explain intervention implementation barriers and enablers. It contains 14 domains (see Table 1) that cover individual, social and environmental and resource factors. The COM-B [9] ‘Capability’ (knowledge and skills), ‘Opportunity’ (factors that lie outside of an individual that mediate behaviour), ‘Motivation’ (individual cognitive processes that direct behaviour) and ‘Behaviour’ model provides a less granular framework for understanding behaviour change and distils the TDF into three interacting key domains [10, 11]. Education and training are two interventions that may serve to act on behaviour, which are considered in this manuscript.

**Table 1:** Mapping of the TDF to the COM-B model [8]

COM-B component	TDF Domain
Capability	Knowledge Skills Memory, attention and decision processes Behavioural regulation
Opportunity	Social influences Environmental context and resources
Motivation	Social/professional role and identity Beliefs about capabilities Optimism Beliefs about consequences Goals Intentions Reinforcement Emotion

While authors have designed individual training interventions considering COM-B [12–14], few have examined whether individual learners have been able to subsequently sustainably implement learning in practice, instead focusing on learners’ immediate perceptions of impact of training on capability and motivation (see for example [15]). No studies to date have considered TDF and COM-B in relation to understanding the barriers and facilitators to sustaining training implementation beyond the initial delivery and implementation phase, or collectively across multiple training programmes and settings. The combination of the two theoretical models allows for exploration of implementation barriers at the individual and organisational level and enables recommendations to be made for potential behaviour change interventions.

The *What Works in dementia education and training? (What Works?)* study aimed to investigate the components of effective dementia training and education, and the barriers and facilitators to its implementation. This was achieved through a systematic literature review [3], a national audit of 386 dementia training programmes in the UK [16], a survey of staff who had completed dementia training and ten organisational mixed-methods case studies in acute hospitals (*n* = 3) [17], mental health Trusts (*n* = 3), care homes (*n* = 3) [18], and primary care (*n* = 1) [19].

## Methods

### Aims

This paper aims to utilise the *What Works?* study data from across these sources to explore the barriers and facilitators to effective dementia education and training implementation in health and social care settings, in the context of the TDF and COM-B model.

### Design

A mixed-methods design [20] combining qualitative and quantitative data from a range of sources of data collected across the study was utilised. This permitted multiple perspectives on the barriers and facilitators to implementation of dementia training to be explored across a broad range of participants and service settings using quantitative methods as well as more in-depth understanding of these factors and their implications using qualitative methods.

### Setting

The research took place in a range of health and social care provider organisations in the UK, who were delivering dementia care. These included acute hospitals, Mental Health Trusts, social care services (care homes), and primary care. Full details of the studies and methods used are published elsewhere [16–19] and an overview is presented below.

### Participants

Participants were recruited differently across the three components of the study; namely the national audit, the staff survey and the case studies. Audit respondents were lead personnel for dementia training within their organisation. They were approached via direct e-mail or via a relevant department within their management services and wide publicity of the survey on social media and at conferences and other events. Staff survey respondents were working in organisations who responded to the audit and had completed at least one of the dementia training programmes reported. They were approached via an e-mail sent from the audit respondent. Ten organisations, that responded to the audit and whose training reflected features of good practice, were selected to take part in case studies. They were approached via sending an e-mail to the audit respondent asking if their organisation would be willing to take part in the case study component. Case study participants were the dementia training lead, the training facilitator(s), ward/unit managers and staff who had attended the organisation’s dementia training.

### Data collection

The data collection took place over three phases. Initially a national audit was administered, which asked organisations to provide details of the types, content and delivery methods of dementia training programmes they provided and questions around barriers and facilitators to implementation. Respondents to the audit were then asked to circulate a survey to staff within their organisation, who had attended the dementia training programme(s) they had reported in their audit response. Finally, the case study sites were selected by examining the audit responses and selecting organisations whose training demonstrated examples of good training practice. The features of good training practice were identified through our initial systematic review [3] and English best-practice training content standards. They included use of face-to-face delivery methods and coverage of at least 75% of the learning outcomes for at least one subject area of the Dementia Core Skills Education and Training Framework [21]. Organisational quality was also checked using publicly available information via Care Quality Commission regulatory reports and NHS Safety Thermometer data. The training audit and staff survey were administered on-line using SNAP Surveys software. They contained fixed and open response questions pertaining to barriers and facilitators to training implementation. In the case study sites, staff who had attended training were asked to complete an on-line or paper-based copy of the Barriers and Facilitators Questionnaire [22], which is based on the Theoretical Domains Framework [8] and open-ended questions on barriers and facilitators faced. The Barriers and Facilitators Questionnaire is a 30-item measure using a 5-point Likert scale (strongly agree to strongly disagree). An average score across items is produced for each domain (knowledge, capabilities, skills, social and professional identity, consequences, motivations, cognitive processes, environmental context, social influences, emotion and action planning). A higher score indicates a domain is perceived as a facilitator and lower score as a barrier (max = 5, min = 1). Individual interviews were conducted with the dementia training lead, training facilitators and managers, and individual interviews or focus groups with staff. Interviews included questions on barriers and facilitators to training implementation (e.g. Managers – “Have you taken any particular steps following staff training to enable it to be embedded in practice?”; Staff – “What aspects of the learning have you/have you not been able to put your learning into practice? What has helped with this/enabled it to happen? OR What has prevented you doing so?”; Trainers – “What do you think has helped your training to be successful and achieve its particular outcomes or indicators?” “Is there anything you’d like to change, or with hindsight, would have done differently?”).

### Data analysis

Quantitative data was analysed using descriptive statistics by SP, SS and CAS. The TDF domains within the Barriers and Facilitators Questionnaire were mapped onto the COM-B model (see Table 1) and a mean score per COM-B domain was calculated across all respondents. Thematic template analysis [23, 24] was used to analyse the interviews and focus groups. Template variant of thematic analysis was selected as it permits a combination of a priori themes relevant to answering a research question, (in this case the themes of barriers and facilitators to training implementation) alongside inductive coding to be used to develop additional themes and sub-themes during analysis. This process leads to development of a coding template. The coding template was developed by CAS, MD, CS, SB and JO who completed collaborative coding of three initial transcripts and discussion of the identified themes. A further six transcripts were then coded to refine the template. This final template was then used to code the remaining data. The qualitative data from the audit and staff survey were also subsequently coded against this template by CAS. Data were then triangulated from across the sources by CAS using the components of the COM-B model and the related components of the TDF.

## Results

**Fig. 1:**
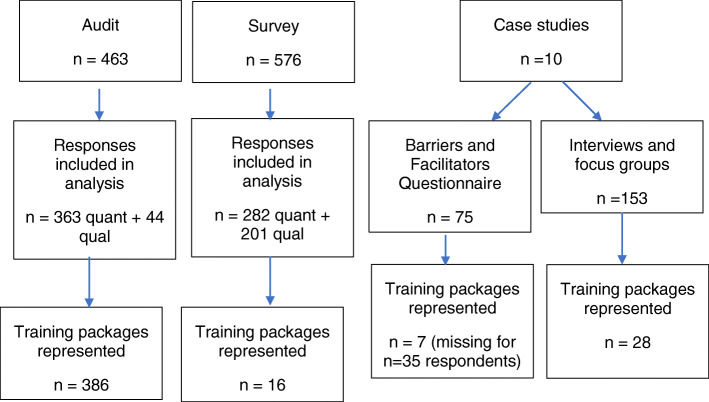
Diagram of data included in analysis

The audit was completed by 436 individuals reporting on 386 training packages. Of these one of the fixed barrier or facilitator responses had been given for 363 packages and qualitative ‘other’ comments were provided on 44 packages. From the Staff Survey, only training packages where at least 10 staff members responded were included in the analysis, leading to 576 surveys eligible for inclusion. Of which 282 of these included responses to the fixed response questions on training implementation and 201 provided qualitative comments, representing 16 different training packages. The responses for some packages were from staff within a single organisation, while others were national programmes and respondents were based in a range of service settings and locations. Barriers and Facilitators Questionnaire data was obtained from 75 respondents and interviews/focus groups were conducted with 153 staff across the 10 case study sites. Please see Fig. 1 for an overview of data included in the study.

Anonymised quotations from participant interviews are presented to illustrate the identified themes, in addition to tables summarising the quantitative data. A code reflecting their site type (AT = Acute Hospital, MHT = Mental Health Trust, CH = Care Home, PC = Primary Care), unique identifying number and participant role and number is shown.

### General training implementation

Of the staff responding to the on-line survey, over 85% felt they had, to some extent, been able to apply their learning in practice and over three-quarters felt supported to some extent or greater, by their organisation to use what they had learned (see Table 2). While this indicates generally positive perspectives on training implementation from this data source, it should be noted that only just over half (55%) felt they had been able to implement learning to a great extent and less than half (49%) felt supported to a great extent by their organisation to do so. This indicates there may be further work many organisations need to do to ensure higher levels of consistent support for training implementation.

**Table 2:** Staff survey responses

Staff survey question n (%)	To a large extent				Not at all
	5	4	3	2	1
To what extent have you been able to use what you have learned from your dementia training in your role? (*n* = 281)	154 (55%)	87 (31%)	31 (11%)	0 (0%)	9 (3%)
To what extent is your organisation supporting you to use what you have learned? (*n* = 282)	137 (49%)	81 (29%)	48 (17%)	11 (4%)	5 (2%)

The following sections will present data according to the components of the COM-B model, presenting data from across the data sources.

### Capability (knowledge; skills; memory, attention and decision processes; behavioural regulation)

Generally, skills and staff capabilities were not perceived as a significant barrier to implementing learning in practice across any of the data sources. Table 3 shows the mean Barriers and Facilitators Questionnaire scores for each COM-B domain It indicates capability domains were overall seen as a facilitator, meaning staff felt they had the requisite knowledge, skills and memory/attention components to implement their training in practice.

**Table 3:** Average Barriers and Facilitators Questionnaire scores by COM-B domain

*N* = 75	C	O	M
Mean score (SD) Range	4.13 (0.48) (3–5)	3.91 (0.57) (2.5–5)	3.78 (0.36) (2.94–4.5)

Significant capability-related facilitators to learning included the use of interactive, group-based learning and the knowledge gained from attending training. Barriers included: training accessibility, acceptability and relevance.

The strongest capability-related facilitator reported in the qualitative data was the use of interactive, face-to-face learning. Case study participants in particular identified this as important with one participant highlighting the impact of having a person with dementia talk to them during their training session.

*Everything he talked about it made sense, because obviously he’s going through it, and obviously we’ve got patients on the ward and there was just certain things about how we should approach somebody like that, and maybe speak to them and care wise as well (AT044 Focus group 2)*

*P1: Yeah, it wasn’t really dry either. There were lots of interactive parts to it wasn’t there?*

*P2: Yeah.*

*P1: Where we’re advising to kind of analyse case studies and put a lot of thought into it what we’d read. So, yeah, we weren’t being spoken at, were we? (MHT062 participants 007 and 008)*

The only staff survey participants to mention the training delivery methods as a facilitator were those who had attended an experiential learning programme, which included engaging in a simulation of what it may be like to live with dementia, demonstrating the high, positive impact of using interactive training methods.

*The fact that I have glimpse of how it may be to experience dementia - this allow me to display sympathy (Staff survey respondent)*

In contrast some case study and audit participants compared their experiences of interactive, face-to-face training with those of self-directed study and e-learning, which were felt to provide inadequate support.

*[E-learning is like] giving people a lot of ingredients and saying go and make, go bake a cake (CH040 Unit Manager)*

*Not all learners are confident with e-learning (Audit respondent)*

Use of self-directed learning with hard copy workbooks also meant training was often abandoned.

*I can’t even remember what I got up to [in training booklet], probably not even half of it"*. *(CH042 Staff Member 032)*

Fifty staff survey respondents noted that improved knowledge and confidence had helped them to implement training in practice, but others hesitated in doing so if training was not seen as relevant to their role or needs, or did not provide new learning. For example, the following respondent commented on knowledge gains in response to the survey question ‘What has helped you to put your learning into practice?’

*My greater knowledge and understanding of dementia (Staff survey participant)*

*One of the things, one of the staff nurses who has been on the training, she felt that maybe it wasn’t, not necessarily pitched at the right level but it kind of, maybe it didn’t recognise and acknowledge that the service has moved on in the last 5 years and … they were still trying to reinforce things that we do, do already … (MHT062 Ward Manager 022)*

### Opportunity (social influences; environmental context and resources)

Opportunity barriers and facilitators were the most frequently identified category across all data sources. Themes included: availability of physical and financial resources, external support, internal support and social influences including organisational culture and leadership. However, the average score for opportunity domains on the Barriers and Facilitators Questionnaires (see Table 3) was the middle score across the three domains and trended towards being a facilitator with a mean score of 3.91.

The most commonly identified opportunity barriers were: lack of resources (including time, financial and competing demands; staffing issues and turnover; and physical, such as ward environments and dedicated training space).

*… there hasn’t been the implementation because of barriers in the workplace – time … they’re stretched anyway in the nursing homes (CH076 Training Facilitator)*

*It’s a difficult situation, because the ward environment isn’t as practical as it probably needs to be for dementia patients and to be as dementia friendly as possible, … It’s kind of, like, learning a lot of that information, but actually not being able to implement everything. (AT066 Staff Member 016)*

Stating that they infrequently supported people with dementia within their role and therefore having little opportunity to implement training in practice, was perceived as a barrier by a number of staff survey respondents.

The availability of external support (other agencies, families) to implement change was considered to impact on opportunity to implement training in the case study sites and by audit and survey respondents.

*Family can sometimes create barriers, if they have their own thoughts on what is best for the person with dementia (Survey respondent)*

*Support from [external] financial providers, Local colleges, [provides the] initiative to share knowledge and enhance practice (Audit respondent)*

A key facilitator of implementation was internal support; supportive management, organisational culture, leadership and peer support for training implementation featured predominantly across all data sources. Management and peer support were cited as important in all but one of the case study sites, and were the highest rated of the facilitators by audit respondents and by many survey respondents.

*The Chief Exec came himself to the carers café and he said “This is fantastic what you’re doing, it’s really great, raising awareness and what can we do to help?” (AT438 Training Lead)*

*Support from Managers and colleagues to reinforce what was learned from training (Staff survey respondent)*

Additional support to implement learning on an ongoing basis, for example through mentorship, supervision and feedback was also an identified facilitator for development of capability.

*I don’t feel like we need to remind people to apply their training, it’s embedded in supervision and the MDT and the way we work and I think our staff are very person centred (MHT068 Ward Manager 042)*

The culture of the organisation and the individual wards/units was identified as crucial to training implementation in half the case study sites and by some survey respondents.

*… people have changed how they approach certain behaviours, think outside the box, maybe question certain policies and procedures and I think it’s embedded into the culture now and staff won’t tolerate certain behaviours in their colleagues. (MHT068 Ward Manager 042)*

*Actually, the crux of it is about the leadership on the ward because if staff are encouraged to share that knowledge when they get back to the shop floor you are more likely to see a change in the culture on the ward. Whereas if you send somebody on training, if they come back to the ward and you don’t have that culture on the shop floor, then that training stays with them but doesn’t get shared and therefore the culture remains (AT044 Trainer 045)*

Leadership for dementia training was also a strong facilitator of implementation of learning in practice in half of the case study sites and cited by several survey respondents.

*… they have revamped … the training department and the training strategy and dementia tier-one and tier-two are in there and also the Trust chief exec … came on the dementia champion training day with a lot of us. (MHT062 Staff Member 006)*

*Positive trust attitude towards dementia care (Staff survey respondent)*

All the organisations taking part in case studies had a designated dementia training lead. They worked proactively and often tirelessly to keep dementia training and practice development on the organisation’s and staff’s agenda.

*… you’re never off. I’m always thinking about stuff. But, if you come into this role because you think it’s just something else you want to do, you can’t, you have got to put in that extra time and effort of wanting to go network. People just don’t invite you to things, you’ve got to put yourself on people’s doorsteps; you’ve got to get yourself known, you’ve got to be proactive (AT044 Dementia Lead)*

### Motivation (role; beliefs about capabilities and consequences; optimism; goals; intentions; reinforcement; emotion)

Motivational barriers and facilitators associated with the training experience included peer learning, incentives and personal values. Motivational domains were the lowest of the three mean scores on the barriers and facilitators questionnaire (see Table 3) (mean score of 3.77), indicating that while they were generally still seen as a facilitator rather than a barrier, this was less so than C and O domains.

A commonly identified theme across the case study sites was the importance of having a skilled training facilitator, who was knowledgeable and could create a memorable and reinforcing learning experience.

*I was feeling very confident with [name]. The way she did the training is very good. (CH042 Staff member Focus Group1).*

Whilst some respondents commented they found mixed groups unhelpful, many staff appreciated training amongst mixed peer groups that included staff from other services or professional roles. This not only supported peer-to-peer learning, but increased understanding and motivation for why practice changes might be needed.

*So, if I explain something about an elderly gentleman walking down a corridor, and a bannister’s cream, the wall’s cream, and the radiator, which isn’t covered, is also cream. He goes on the radiator, by the time he’s got to the end, he’s got a third-degree burn. And so many times, I’ve had nurses from the Burns Unit say ‘Oh, we’ve got somebody in at the moment, we only nursed somebody like that the other week!’ And it tells the rest of the room ‘this is real.’ (AT438 Trainer 021)*

Providing incentives for attending training (e.g. during paid working hours, badges/certificates) facilitated motivation as identified by case study and audit respondents. Staff in two case study sites identified how they became demotivated and did not engage with training when expected to complete it in their own time. Designated time for dementia training was identified as a motivating facilitator for 159 of the audit training packages.

*You can’t just expect them to pitch up and not be paid. (CH040 Unit Manager 020)*

*And the company provides the time and pay, we paid them today [to attend training] (CH076 Unit Manager)*

Personal values such as a desire to learn and improve care practice motivated staff to engage in behaviour change and put learning into practice. Training was discussed as a mechanism for reinforcing good practice.

*I think registered nurses, some of our newer nurses, our overseas nurses especially, they are really keen to learn, they have a real thirst for knowledge and a thirst for learning. So it is actually, it’s really really nice (AT438 Ward manager 022)*

Staff who were disinterested in learning or dementia care, or who had low morale due to issues such as staff shortages, lacked motivation to engage in training and thus to modify their behaviour to put it into practice.

*… we have done a couple of [sessions] which people need to attend for their revalidation. I remember doing it and one Doctor fell asleep! It’s demoralising, really,’ (AT044 Dementia Lead)*

And where colleagues failed to appreciate the need for training, participants identified this as a barrier to behaviour change.

*From carer point of view once again it’s a classic case of if someone’s been here for twenty odd years it’s “Why do I have to go on the dementia training? I went on it ten years ago.” (AT438 Ward Manager 022)*

However, training could also create feelings of empowerment and confidence towards taking a leading role in practice change.

*… people talk about that bottom-up kind of change now and I think that is a bit of a slogan personally. But, that’s ultimately what it is in effect but a less fancier version. (MHT029 Ward Manager 02)*

## Discussion

This is the first study to apply the TDF and COM-B model to staff behaviour change and application of learning in practice across a range of service types, settings and staff. In total our data includes the perceptions of over 940 staff, representing over 380 training packages across hundreds of health and social care provider organisations. Of the COM-B components, staff capability issues reflected the lowest level of barriers to behaviour change. The use of interactive, face-to-face learning and the increased knowledge and skills that staff felt interactive dementia training provided were strong facilitators to behaviour change. However, where training was self-directed (e.g. e-learning/workbooks) and not tailored to their role or was seen to provide no new knowledge, this was a barrier to learning, and hence impeded behaviour change in practice. Opportunity to apply learning in practice was the most commonly cited barrier/facilitator, and lack of resources (financial, time, staffing, environmental) was consistently identified as a key barrier to changing practice. External and internal support for implementing training as well as a supportive organisational culture and leadership for dementia training were key facilitators. Motivational facilitators included delivery by a skilled facilitator, opportunities for peer learning and incentives for completing training. Personal and colleagues’ values and attitudes towards dementia care and training could be barriers or facilitators.

In this study, staff consistently reported the use of interactive, face-to-face learning methods as being most effective and some identified passive methods such as e-learning and self-directed workbooks as barriers to learning, as part of behaviour change. Training delivered online has the potential to reach a vast workforce using limited resources and thus is often the method of delivery of choice in healthcare. While research suggests e-learning may not be less effective for knowledge acquisition than face-to-face delivery in healthcare [25–27], impact on practice behaviours and patient outcomes has largely not been evaluated [28]. Given the findings of this study, health and social care providers should consider how they can include interactive and group-based learning within their dementia training portfolio, to optimise the potential for behaviour change.

Staff working in health and social care services come from a variety of backgrounds with differing experiences in terms of education, training and years working in their role. These along with other individual factors can influence confidence, attitudes and motivation for learning. This study found that some staff demonstrated a personal desire for learning and even small rewards for attending training could provide additional motivation. Researchers have indicated that a range of intrinsic and extrinsic factors affect personal motivation for continuing professional development among healthcare professionals [29–31]. These include: the desire to provide quality care, career progression, and compliance with mandatory requirements. Furthermore, specific types of motivations lead to engagement in particular types of learning activities. There is a need to tailor educational provision in this context because, although mandated training may increase completion rates, it does not automatically motivate staff to apply knowledge in practice. It is essential that staff are provided with adequate time to accommodate training and assimilate learning, and that their experience is enhanced through appropriate design, delivery and inclusion of content which supports their practice [32]. Continued support for staff to implement training through mentorship and empowerment was also perceived to support motivation, a finding mirrored in other recent research [5, 33].

Skilled facilitation was identified as motivating staff to apply learning in practice in this study, and this reflects the wider literature on facilitation within health and social care education. For example, crucial facilitation skills include availability, approachability, flexibility and good communication (such as provision of feedback) and perceived expertise in the subject area [34–37]. Appointing dementia training facilitators who can demonstrate these qualities is an essential consideration for care provider organisations.

Our findings that organisations struggled with resources such as time, finances and availability of staff, in order to support staff to change their behaviour through implementation of learning, are commensurate with literature around health and social care globally [38–40]. It can be challenging to prioritise training attendance and subsequently support staff to implement learning in practice. However, strong management support and robust dementia leadership were almost globally identified as essential components for creating the opportunity for widespread uptake of dementia training and its implementation in practice in this study. The important role that management support, organisational culture and leadership play in implementation of training is highlighted throughout the health and social care research literature [41–44] and should be considered a priority for organisations investing in dementia training. Support needs to be visible at all levels of the organisation, with service leaders and managers encouraged to prioritise and champion dementia training and its implementation.

This study is the first to explore application of the TDF and COM-B model to understanding barriers and facilitators to implementing dementia training in practice (i.e. to staff behaviour change) across a range of health and social care organisations, individuals working in these settings and dementia training programmes. While the audit, survey and case studies may not have provided a representative sample of care and training providers or staff working within these settings, respondents did come from a range of services. We were unable to include organisations that provided no dementia training to their staff owing to the design of our online audit, and thus cannot evaluate the associated barriers to commencing training delivery. This is a recommended avenue for future research. Likewise, we did not include the perspectives of people affected by dementia in discussing barriers and facilitators to training implementation. The case study sites were recruited as exemplars of good practice, therefore additional barriers to implementation may exist in organisations that are less successful in this area. The views of some staff may be repeated across the survey and case study data collection.

## Conclusions

Evaluations of dementia training which only consider content and methods of training delivery are unlikely to lead to successful staff behaviour change. The TDF and COM-B model have provided a useful framework for identifying broader relevant factors. Health and social care provider organisations may benefit from utilising these to support staff in relation to the range of capability, opportunity and motivational factors that need to be addressed for staff to successfully implement their learning into practice.

## Data Availability

The datasets generated and/or analysed during the current study are not publicly available but are available from the corresponding author on reasonable request.

## Abbreviations

AT: Acute Hospital
CH: Care Home
COM-B model: Capacity, Opportunity, Motivation, Behaviour model
MHT: Mental Health Trust
PC: Primary Care
TDF: Theoretical Domains Framework
